# Supplementary material for: Activin receptor ALK4 promotes adipose tissue hyperplasia by suppressing differentiation of adipocyte precursors
Source: J Biol Chem. 2022 Nov 18;299(1):102716. doi: 10.1016/j.jbc.2022.102716 (PMC9758429; doi:10.1016/j.jbc.2022.102716)
Supplement: Supporting information [file mmc1.docx]

**Supplementary Information**

**Supplementary Table S1. Primer sequences of mouse genes**

| **mRNA** | **Forward primer (5' -> 3')** | **Reverse primer (5' -> 3')** |
| --- | --- | --- |
| *Alk4* | AGAGGGTGGGGACCAAAC | TGCTTCATGTTGATTGTCTCG |
| *Alk7* | GCTTTCCATAGCGAGTGGTC | ATCTCGGTGAGCAATAGCAG |
| *Adipoq* | GAAGCCGCTTATGTGTATCGC | GAATGGGTACATTGGGAACAGT |
| *Cd24a* | ACTCAGGCCAGGAAACGTCTC | AACAGCCAATTCGAGGTGGAC |
| *Cebpα* | CAAGAACAGCAACGAGTACCG | GTCACTGGTCAACTCCAGCAC |
| *Fabp4* | ACACCGAGATTTCCTTCAAACTG | CCATCTAGGGTTATGATGCTCTTCA |
| *Gdf3* | ATGCAGCCTTATCAACGGCTT | AGGCGCTTTCTCTAATCCCAG |
| *Inhba* | ATCATCACCTTTGCCGAGTC | TCACTGCCTTCCTTGGAAAT |
| *Inhbb* | GATCATCAGCTTTGCAGAGACA | TGCCTTCATTAGAGACGAAGAA |
| *Leptin* | GAGACCCCTGTGTCGGTTC | CTGCGTGTGTGAAATGTCATTG |
| *Mki67* | ATCATTGACCGCTCCTTTAGGT | GCTCGCCTTGATGGTTCCT |
| *Pdgfra* | TCCTTCTACCACCTCAGCGAG | CCGGATGGTCACTCTTTAGGAAG |
| *Pdgfrb* | AGACACTGGGGAATACTTTTGTG | CGGCCCTAGTGAGTTGTTGT |
| *Pparγ* | GGAAGACCACTCGCATTCCTT | GTAATCAGCAACCATTGGGTCA |
| *Tbp* | ACCCTTCACCAATGACTCCTATG | TGACTGCAGCAAATCGCTTGG |
| *Ywhaz* | CAGTAGATGGAGAAAGATTTGC | GGGACAATTAGGGAAGTAAGT |
| *Zfp423* | GTCACCAGTGCCCAGGAAGAAGAC | AACATCTGGTTGCACAGTTTACACTCAT |

**Supplementary Table S2. Primer sequences of human genes**

| **mRNA** | **Forward primer (5' -> 3')** | **Reverse primer (5' -> 3')** |
| --- | --- | --- |
| *Alk4* | ACTGGTGGCAGAGTTATGAGG | GCATACCAACACTCTCGCATC |
| *Alk7* | AACATAACACTGCACCTTCCAA | CTCCATGGGTCCAAGTTTTG |
| *AdipoQ* | GTGATGGCAGAGATGGCAC | GCCTTGTCCTTCTTGAAGAG |
| *Cebpα* | TATAGGCTGGGCTTCCCCTT | AGCTTTCTGGTGTGACTCGG |
| *Fabp4* | CCACCATAAAGAGAAAACGAGAG | GTGGAAGTGACGCCTTTCAT |
| *Leptin* | TTGTCACCAGGATCAATGACA | GTCCAAACCGGTGACTTTCT |
| *Pparγ* | GACAGGAAAGACAACAGACAAATC | GGGGTGATGTGTTTGAACTTG |
| *TBP* | GAACATCATGGATCAGAACAACA | ATAGGGATTCCGGGAGTCAT |
| *YWHAZ* | GATCCCCAATGCTTCACAAG | TGCTTGTTGTGACTGATCGAC |

**Supplementary Table S3. Antibodies used for immunoblotting**

| **Antibody** | **Dilution** | **Manufacturer, Catalogue number** |
| --- | --- | --- |
| CEBPα | 1:1000 | Cell Signaling Technology, USA, #8178 |
| HSP90 | 1:5000 | Cell Signaling Technology, USA, #4877 |
| PPARγ | 1:1000 | Cell Signaling Technology, USA, #2443 |
| pSMAD2 | 1:1000 | Cell Signaling Technology, USA, #3108 |
| SMAD2 | 1:1000 | Cell Signaling Technology, USA, #5339 |
| pSMAD3 | 1:500 | Cell Signaling Technology, USA, #9520 |
| SMAD3 | 1:500 | Cell Signaling Technology, USA, #9523 |
